# Frequency of Tonsilloliths in Panoramic Views of a Selected Population in Southern Iran

**Published:** 2015-06

**Authors:** Janan Ghabanchi, Abdolaziz Haghnegahdar, Leila Khojastehpour, Ali Ebrahimi

**Affiliations:** 1 Dept. of Oral and Maxillofacial medicine, School of dentistry, Shiraz University of Medical Sciences, Shiraz, Iran;; 2 Dept. of Oral &Maxillofacial Radiology, School of dentistry, Shiraz University of Medical Sciences, Shiraz, Iran;; 3 Undergraduate Student, Student Research Committee, School of Dentistry, Shiraz University of Medical Sciences, Shiraz, Iran;

**Keywords:** Tonsilloliths, Panoramic radiographs, Palatine tonsil, Radiopaque lesions

## Abstract

**Statement of the Problem:**

Tonsilloliths are relatively common clusters of dystrophic calcified material that form in the tonsillar crypts, mostly the palatine tonsils. Although they may be asymptomatic, some cause halitosis, cough, dysphagia, and foreign body sensation, as well as otalgia. Since tonsilloliths can be detected on panoramic views as radiopaque lesions, and misdiagnosis may lead to wasting time and cost, dentist should be familiar with radiographic characteristics of this type of calcification.

**Purpose:**

This study was conducted to determine the prevalence and the pattern of distribution of tonsilloliths on panoramic radiographs.

**Materials and Method:**

This cross-sectional study was based on 2000 panoramic radiographs from 1030 female and 970 male aged 6-75 years old evaluated for the presence and pattern of tonsillolithiasis, between 2011 and 2013 in Shiraz, Iran. Chi–square test and odds ratio were used to evaluate the relationship between tonsillolithiasis and gender. *p*< 0.05 was considered as statistically significant.

**Results:**

Out of the 2000 individuals, 101 cases (5.05%) had tonsilloliths on panoramic radiographs out of which 61 were male (60.4%) and 40 were female (39.6%), with age range of 18 to 65. Forty patients (39.6%) had both left and right sides involved, 25 of tonsilloliths (24.75%) were located on the right and 36 on the left side (35.65%). Men were more likely to develop tonsilloliths (*p*= 0.014).

**Conclusion:**

Tonsilloliths are not very common finding and can be detected on nearly 5.05% of panoramic radiographs. Most of the cases are unilateral with a diameter less than 2mm.

## Introduction

Tonsilloliths, also known as tonsil stones or tonsillar calculi are uncommon calcified structures that develop in enlarged tonsillar crypts, due to a rare form of dystrophic calcification.[1-2] These stones are composed of calcium salts such as hydroxyapatite or calcium carbonate apatite, oxalates, and other magnesium salts and ammonium radicals. They are usually of small sizes and their formation etiology and pathogenesis are unknown. Some researchers believe that tonsilloliths can be related to lithiasis in other regions of the body.[3]

**Figure 1:**
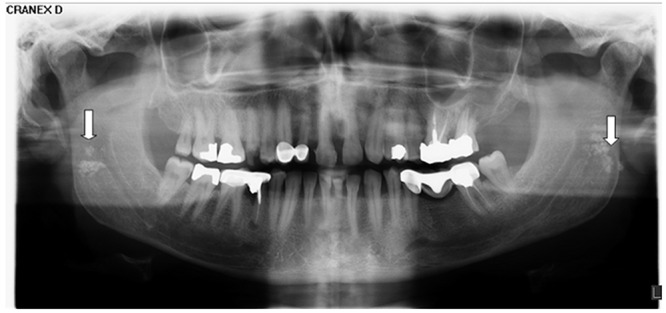
A case of bilateral tonsilloliths accompanied with unilateral elongated styloid process on the right

Tonsillar calculi primarily involve the palatine tonsil and may be asymptomatic, hence- they are usually discovered incidentally on panoramic radiographs. However, non-specific symptoms such as chronic halitosis, foreign body sensation, dysphagia, pain in ear, and irritable cough or foul taste are reported as the accompanying symptoms.[4] Distinguishing tonsilloliths from- other radiopaque lesions of the jaws may be difficult especially in asymptomatic patients. Differential diagnosis includes malignancies, lymph node and salivary gland calcifications, phleboliths, calcified granulomas, and scrofula. Certain diseases such as tuberculosis, tertiary syphilis, deep fungal infections, foreign bodies, isolated bone or cartilage derived from embryonic rests, or an elongated styloid process could also be suspected.[5-6] Misdiagnosis may impose fear and huge stress on the patient in addition to inappropriate treatments. Having knowledge about the incidence, anatomic location, radiographic appearance, usual size and number of tonsilloliths help save time and cost. Radiographs are the first diagnostic tool in order to detect these radiopaque lesions in the jaws. On panoramic views, tonsillitis appear as single or multiple defined radiopacities on the mandibular ramus, in the region where the dorsal surface of the tongue crosses the ramus in the palatoglossal or glossopharyngeal air spaces (Figure 1).[4, 7] Although these radiopacities are usually seen in a limited area (palatine tonsil), they may be misdiagnosed with other calcified lesions on the image. So, tonsilloliths must be considered as the first differential diagnosis for multiple ill-defined radiopacities detected on the palatal uvula and ramus.[4] Tonsilloliths may be superficially located in tonsillar crypts, seen as a yellowish calcified mass or more deeply located and felt as an enlarged hard lesion.[8] Large-sized tonsilloliths tend to be single while smaller ones are usually multiple.[9] Since they may be present either unilaterally or bilaterally in an image, ghost images should be ruled out in a panoramic radiograph.[10] Finally, it is important to note that tonsilloliths rarely complicate the patient’s condition; although sporadically, peritonsillar abscess and trismus may appear because of the penetration of tonsillar capsule.[11] In elderly patients, pulmonary complications may be induced secondary to large tonsilloliths aspiration.[1] Some studies demonstrated that tonsilloliths are related to halitosis and tonsillar abscess.[12]

Literature review showed that the detection rate of tonsilloliths was under 25%, however some believe otherwise.[13-14] Histological examinations revealed 8% prevalence of these concretions.[4]

Babu *et al.* used panoramic view to present an unusual bilateral and asymptomatic case of tonsilloliths.[4] Misirlioglu *et al. *reported that cone beam computed tomography (CBCT) should be used in diagnosis of all bilateral cases of tonsilloliths found in panoramic views, to eliminate unilateral cases with ghost shadows, mistaken as bilateral forms.[15]

Fauroux *et al.* reported the prevalence of tonsilloliths to be 24.6% in 150 consecutive CT examinations.[16]Aghdasi *et al.* evaluated the panoramic view of 966 patients and reported an incidence of 4.9 % for tonsilloliths. They concluded that tonsilloliths are age-related but sex-independent. Most of the cases were left sided and 48.9% were bilateral.[17]

As mentioned before, tonsilloliths can cause various oral and maxillofacial symptoms which unfortunately some dentists are not familiar with. To the best of authors’ knowledge, no epidemiologic study has been conducted on frequency of tonsillolithiasis in this part of the country. This study was aimed to evaluate the prevaLence and distribution pattern of tonsilloliths on panoramic views in southern Iran. 

## Materials and Method

In this cross-sectional study, 2000 panoramic views (1030 female, 970 male, age 6 to 75 years old) of patients referring to a private clinic of maxillofacial radiology, between 2011 and 2013, were retrieved and re-evaluated for presence of tonsilloliths.

Panoramic radiographs were acquired using a digital panoramic *CRANEX® D SOREDEX* device (Finland; 57-85 kVp, 10mA). All panoramic views of acceptable quality were recruited in the study. The presence of tonsillar calculi was evaluated on panoramic radiographs by two calibrated oral radiologists and a post graduate student, using the criteria reported by Ram *et al.* that mentioned tonsilloliths as radiopaque nodular mass, or masses piled up on the mandibular ramus and soft palate.[12]

 Unilateral or bilateral occurrence, number, and size of the stones were recorded. The cases with tonsilloliths were divided to subgroups according to both size (less or more than 2 mm) and number (less or more than 5).

Since the radiographs had been taken for other dental purposes, no extra dose was exposed to the patients and no name or personal information of patients was recorded in reports and figures, hence, there was no ethical limitation to conduct the study.

Data were described using frequency and percentage. Chi-square test, odds ratio (OR) and 95% confidence interval (95% C.I) were used to assess the relationship between gender and prevalence of tonsilloliths. *p*< 0.05 was considered statistically significant.

## Results

Out of 2000 individuals, 101 (5.05%) were judged to have tonsilloliths on panoramic radiographs (Table 1).

**Table 1:** Overall findings of the research

	**Number**	**%**
Studied radiographs	2000	100
Female	1030	51.5
Male	970	48.5
Radiographs with tonsilloliths	101	5.05

Among them, 61 were male (60.4%) and 40 were female (39.6%) aged between 18-65 years. In 40 patients (39.6%) both left and right sides were affected including 25 on the right side (24.75%) and 36 on the left side (35.65%) (Table 2). Compared to women, men were found to be 1.70 times more likely to develop tonsilloliths (OR=1.70 / Y.95CI: (1.10- 2.50), *p*= 0.014).

The size of stones varied on panoramic radiographs and was less than 2 mm in 66 cases (65.35%) and more than 2mm in 35 cases (34.65%) (Table 2).

**Table 2:** Distribution of tonsilloliths

	**Tonsilloliths**	**Male**	**Female**	**Bilateral**	**Unilateral**		**Size**		**Multiplicity**	
					**Right**	**Left**	**< 2 mm**	**≥2 mm**	**< 5**	**≥5**
Number	101	61	40	40	25	36	66	35	63	38
%	5.05%	60.4%	39.6%	39.6%	24.75%	35.65%	65.35%	34.65%	62.38%	37.62%

**Figure 2:**
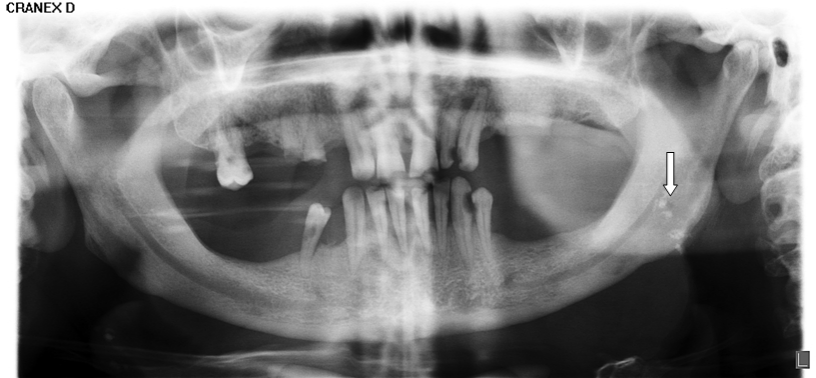
A case of multiple tonsilloliths on the left side

The tonsilloliths were observed in different shapes such as round, oval, and irregular structures. The number of calcified masses varied from less than 5 in 63 cases (62.38%) to more than 5 in 38 cases (37.62%) (Figure 2). The calcification (elongation) of stylohyoid ligament accompanied with tonsilloliths in 37 cases (36.63%).

## Discussion

Despite the fact that the cause and pathogenesis of tonsilloliths are not clearly known, the researchers believe that unresolved tonsillitis is the main factor. Meanwhile, many other authors have suggested that tonsilloliths developed as a result of stasis of the saliva in the efferent ducts of the accessory salivary glands secondary to mechanical obstruction arising from post-tonsillectomy scars or chronic inflammation.[13] Frequent episodes of inflammation may cause fibrosis at the openings of the tonsillar crypts, causing bacterial and epithelial debris accumulation which leads to retention cysts formation. Subsequent calcification due to deposition of inorganic salts and enlargement of the formed concretion take place gradually. Diagnosis of tonsilloliths will be confirmed through clinical presentation, examination and imaging. In physical examination, enlargement and hardening of the tonsil is a common finding. On extra-oral radiographs, these calcifications may be confused with other lesions such as tooth, foreign bodies, salivary gland and lymph nodes calcifications or stylohyoid ligament elongation.

Panoramic radiograph supplemented by a CT scan is helpful in diagnosis of such lesions.[2] CBCT images from both sides of the jaw are useful to determine the exact locations of these calcifications.[18]

Sialoliths, lymph node calcification and phleboliths are included in differential diagnosis of tonsilloliths more frequently. Sialolithiasis (salivary calculi or salivary stones) is a calcified mass formed within a salivary gland, frequently in the duct of the submandibular gland (Wharton's duct). Parotid gland is less commonly involved; rarely do the sublingual gland or minor salivary glands develop salivary stones.[19] Sialoliths are usually accompanied with pain and swelling of the affected salivary gland. On coronal and axial tomographic images, parotid gland calcification is located on the exterior of the mandible.

Phleboliths are usually multiple and have a clustered distribution. Frequently they are associated with heamagioma. Phleboliths also tend to be lamellated, while tonsilloliths are simply opaque.

The submandibular region is the common site for calcification of lymph nodes which may be associated with tuberculosis or other granulomatous diseases.[18] Differential diagnosis of atherosclerosis in carotid arteries from tonsilloliths may be of interest because of cardiovascular risk associated with this entity. Fortunately, the anatomic location of these two calcifications is clearly different. Tonsilloliths are usually superimposed over the ramus and dorsum of the tongue, while carotid atherosclerosis is usually found at the level of third and forth cervical vertebrae.

In this study, we used panoramic views as screening tool. Although newer modalities of imaging such as CBCT may be more accurate and reliable in diagnosis of tonsilloliths; they could not be employed in such epidemiologic studies, because they are associated with high cost and relatively high doses of radiation to cases. Panoramic view is an easy-access imaging modality, with low-dose radiation, used in most of dental procedures. We used those panoramic radiographs, prepared for other dental purposes, without exposing the patients to unnecessary extra doses.

In a case report, Protnof *et al.* showed that tonsilloliths can appear as multiple opacities on a panoramic image.[20] Also Guevara and Mandel reported the same radiographic finding and mentioned that tonsilloliths were superimposed on the mandibular ramus.[18] These reports agree with choosing the panoramic view as a survey tool for diagnosis of tonsilloliths.

Fauroux *et al.* evaluated 150 CT images and showed that 37 patient (24.6%) had palatine tonsilloliths.[16] Oda *et al.* detected two different rates of prevalence for tonsilloliths in 482 pairs of consecutive CT and panoramic radiographs, 46.1% in CT scans and 7.3% on panoramic views. In the current study, CT scan images were not used but approximately 5% of panoramic radiographs showed tonsilloliths which can be comparable with what they reported on panoramic views; but very different from their CT scan findings. This significant difference may be attributed not only to the characteristics of two different modalities but also to the different indications of these techniques. Despite the fact that CT scan is more efficient in locating tonsilloliths, it is wiser to use panoramic views as screening tool in such studies to prevent bias.

It is noteworthy that there was no case of tonsilloliths under the age of 18 or over 65 in the current study. This may show the age range of this condition but more probably might be due to the unequal distribution of samples in different age groups in the current research. Future studies are suggested to evaluate the prevalence of tonsilloliths with equal samples in different age groups in order to determine the relationship between age and tonsillolithiasis. Finally, the importance of hygiene condition and epidemiologic pattern of diseases of ear, nose and throat in different areas should be considered to explain the discrepancy in rate of tonsilloliths in different studies. If tonsillitis is considered as a major etiology for tonsillolithiasis, the above-mentioned factors will definitely play a role. 

## Conclusion

The prevalence of tonsilloliths in the studied population was 5.05%. Most of the cases were unilateral with sizes more than 2 mm and number of less than five in an individual. 
